# Comparing the measurement equivalence of EQ-5D-5L across different modes of administration

**DOI:** 10.1186/s12955-015-0382-6

**Published:** 2015-11-26

**Authors:** Brendan Mulhern, Hannah O’Gorman, Neil Rotherham, John Brazier

**Affiliations:** Centre for Health Economics Research and Evaluation, University of Technology Sydney, Centre for Health Economics Research and Evaluation, Sydney, Australia; School of Health and Related Research, University of Sheffield, Sheffield, UK; Exco InTouch, Nottingham, UK

## Abstract

**Background:**

Interest in collecting Patient Reported Outcomes using electronic methods such as mobile phones has increased in recent years. However there is debate about the level of measurement equivalence between the traditional paper and newer electronic modes. Information about the acceptability of the electronic versions to respondents is also required. The aim of this study is to compare the equivalence of delivering a widely used generic measure of health status (EQ-5D-5L) across two administration modes (paper and mobile phone).

**Methods:**

Respondents from a research cohort of people in South Yorkshire were identified, and randomly allocated to one of two administration modes (paper vs. mobile phone) based on stratifications for age and gender (and across a range of self-reported health conditions). A parallel group design was used where each respondent only completed EQ-5D-5L using one of the modes. In total, 70 respondents completed the measure in the mobile phone arm, and 66 completed the standard paper version. Follow up usability questions were also included to assess the acceptability of the mobile version of EQ-5D-5L. Measurement equivalence was compared at the dimension, utility score and visual analogue scale level using chi square analysis and ANOVA, and by comparing mean differences to an estimated minimally important difference value.

**Results:**

Response rates were higher in the mobile arm. The mean EQ-5D-5L utility and VAS scores, and the frequency of respondents endorsing individual EQ-5D-5L dimension response levels did not significantly differ across the administration modes. The majority of the mobile arm agreed that the mobile version of EQ-5D-5L was easy to complete, and that the phone was easy to use, and that they would complete mobile health measures again.

**Conclusions:**

Completing health status measures such as EQ-5D using mobile phones produces equivalent results to more traditional methods, but with added benefits (for example lessening the burden of data entry). Respondents are positive towards completing questionnaires using these methods. The results provide evidence that electronic measures are valid for use to collect data in a range of settings including clinical trials, routine care, and in health diary settings.

## Background

The use of electronic methods such as laptops, tablets and mobile phones to collect Patient Reported Outcome Measure (PROM) or Clinical Outcome Assessment (COA) data has increased substantially in recent years. Technology advances and the increased prevalence of electronic device ownership around the world [1] means that electronic versions of COA (eCOA) can be used in a wide range of areas including clinical trials, routine health care and clinical settings, and also in the general population as, for example, health diaries that enable the assessment of change in health over time. Furthermore, the potential for extending the use of eCOA given the ongoing increase in the use of mobile devices is large.

Electronic methods can be useful for collecting sensitive data, and reduce the burden of data entry which is done automatically [2, 3]. They are also potentially more convenient to patients as they can be completed at a time that suits them (and this can be recorded automatically) which also has benefits in clinical settings where data can be collected to inform consultations [4]. However paper versions have been seen as the ‘gold standard’ as the majority of existing COAs were developed and validated using this format. COAs may have to be adapted for administration using electronic methods (for example by changing the question layout or reducing the number of questions per page), and subsequently there has been debate about the level of measurement equivalence of data collected using different administration modes. The most recent Food and Drug Administration (FDA) guidance [5] specifies that the measurement properties of eCOA used to support labelling claims should be comparable across modes. In a recent meta-analysis of the comparability of electronic (via computer or PDA) and pencil and paper COA administration, Gwaltney et al. [6] found extensive evidence for equivalence across the modes. Other reviews have also provided evidence for equivalence [7], but also mixed results in terms of which produces the highest quantity and quality of responses [8]. Bushnell et al. [9] compared Personal Digital Assistant (PDA) and paper administrations of EQ-5D in an irritable bowel syndrome population finding no mean difference between the index scores and a high level of reliability across the administrations.

As part of the process of validation of the new technology available, it is important that equivalence between modes is demonstrated, and recommendations for research in this area have been published [10]. It is also important to understand the usability of the technology and the adapted measures. Work investigating the delivery of COAs using PDA’s is widespread, and research investigating the administration of COA using every day mobile devices such as smartphones and tablet computers is increasing. Extending work in this area will support the development of, for example, applications containing outcome measures that could be downloaded onto an individual’s phone or tablet and completed at a time suitable to them. This has benefits for both clinical trials and the more general use of health assessment tools amongst the wider population.

This study aims to add to this literature by comparing the administration of a widely used generic COA (the EQ-5D-5L) between paper and mobile phone administration modes. The primary objectives of the project are:

1. To investigate the equivalence of EQ-5D-5L data collected using different modes of administration (paper vs. mobile device with a touchscreen).
2. To examine the usability of the mobiles phones used to collect data.

## Methods

### Measures and questions used

#### EQ-5D-5L

EQ-5D-5L [11] was used as the key outcome measure in this study, with the administration of both the health descriptive system and visual analogue scale (VAS) compared across the modes. The EQ-5D-5L is an updated version of the three level EQ-5D (EQ-5D-3L) [12], and assesses health status across five dimensions (mobility, self-care, usual activities, pain/discomfort and anxiety/depression) each with five response levels (none, slight, moderate, severe and extreme/unable). The VAS assesses self-reported health status on a 0 to 100 scale where 0 is equivalent to worst imaginable health and 100 is equivalent to the best imaginable health.

The EQ-5D-3L descriptive system has an associated utility ‘value set’ based on the preferences of the general population that allows the measure to be used in the calculation of Quality Adjusted Life Years (QALYs) for use in the economic evaluation of health interventions, and is the measure accepted by reimbursement bodies such as the United Kingdom National Institute of Health and Care Excellence (NICE) and the Australian Pharmaceutical Benefits Advisory Committee (PBAC) for this purpose [13]. The UK value set was developed using the preference elicitation technique Time Trade Off (TTO), and ranges from 1 (for the ‘best’ health state described with no problems on each dimension (11111))–0.594 (for the worse health state with extreme problems (33333)). On this scale, 0 is equivalent to dead, and negative values given to states perceived as worse than being dead (following modelling of the TTO data) [14].

The EQ-5D-3L value set can be mapped onto EQ-5D-5L health states where the best and worst health states are anchored to the EQ-5D-3L utility scale [15]. This ‘crosswalk’ value set is used in the analysis carried out in this study and allows for an assessment of equivalence across modes using a value set directly linked to the EQ-5D-3L.

EQ-5D-5L was used in this study as it is a widely used, concise and validated generic COA that can be used both with the general population and across patient groups. Furthermore, the increasing use of EQ-5D-5L both in cost effectiveness analysis (as part of trials and other studies), population surveys and in routine healthcare settings mean that it is an instrument that can be widely administered using a range of administration modes. Therefore evidence regarding equivalence across modes is important, both for providing evidence for the use of the different modes and equivalence of responses, and also for the use of EQ-5D-5L.

The EQ-5D-5L and VAS were adapted for delivery via mobile technology by the project team (see online supplement 1), with the final version agreed by the EuroQol Group (the EQ-5D-5L copyright holders). The paper version was matched with that recommended for use by the EuroQol Group. In contrast to the paper version, it is recommended that each of the five dimensions appear on a separate page. Slight adaptations to the instructions were made to reflect the fact that a touchscreen was been used. The VAS was answered by pressing on the relevant number on the scale, and the number automatically appeared in the answer box, and could be changed before proceeding. The paper VAS requires respondents to fill in the relevant number in the available box. Each question had to be answered before proceeding, and respondents were able to scroll back to change their answers during the completion of the survey, but not once they had finished the questionnaire. Respondents could stop during the completion of the EQ-5D-5L by just exiting the application containing the measure on the phone, but we could not record this information. Due to the nature of the data collection process, questions on the paper version were not compulsory and changes could be made at any time during the completion of the overall questionnaire.

#### Demographic and wellbeing questions

A range of demographic and health questions were collected from all respondents using a paper questionnaire (that was appended to the EQ-5D-5L for the paper arm, and completed as a separate questionnaire following the EQ-5D-5L for the mobile arm). This included questions about age, gender, marital status, education, and long standing health conditions. The four subjective wellbeing and satisfaction questions used by the Office of National Statistics (ONS) as part of the household survey [16] were also collected, with the addition of an extra question. The questions examined health satisfaction, life satisfaction, happiness and anxiety yesterday, and whether life is perceived as worthwhile on a 10 point scale from ‘not at all’ (0) to ‘completely’ (10).

#### Usability questions

Questions investigating the usability and acceptability of the mobile phones were also included. These investigated whether respondents would use phones to complete COAs again, whether the questionnaire was easy to complete and read, whether the touchscreen could be used, and also the usability of the questionnaire (see online supplement 2). Paper arm respondents were asked whether they would complete COAs such as EQ-5D-5L using a mobile phone.

### Study design

In this study a parallel groups design was used, with respondents randomly allocated to either the mobile (*n* = 100) or paper (*n* = 100) completion arm. The paper questionnaire and mobile device (if relevant) were then sent to the respondents. Detailed instructions about how to access the mobile questionnaire, and the order in which the questions should be completed, were included. After completion, respondents were asked to return the questionnaire and mobile device to the study team using a freepost envelope provided. If there was no response, or if the mobile questionnaire was completed but the device was not returned, then a follow up email (if possible) or letter was sent. This procedure was approved by the School of Health and Related Research, University of Sheffield, ethics committee.

### Sampling and recruitment

Participants in this study were recruited from the Yorkshire Health Study (YHS) [17] between February and September 2014. The YHS is a large scale longitudinal study collecting information over two waves about the health of residents in Yorkshire and Humberside areas of the United Kingdom. The first wave contains records for 27,806 individuals (2010–12), aged between 16 and 85 from South Yorkshire. As part of the survey, respondents are asked to indicate whether they are willing to take part in further research projects, and respondents in this study were identified from those aged over 18 who agreed to be contacted. Eligible respondents were identified and stratified across five age groups (18–30; 31–40; 41–50; 51–60 and 60+) and gender. A random sample of 200 respondents across the age groups and who self-reported a range of health conditions was then selected and randomly allocated to each arm.

### Analysis

The aim of the analysis was to assess the acceptability and usability of the mobile EQ-5D-5L, and compare the scores produced across different samples, in line with the recommendations of Coons and colleagues [10] for comparing COA equivalence. Proportion differences across the various demographic indicators and EQ-5D responses were tested using chi square analysis. ANOVA difference testing was used to assess the equivalence of EQ-5D-5L utility and VAS scores across the arms. The magnitude of the mean difference between the utility and VAS scores was also assessed in comparison to an estimated value for the minimally important difference (MID) of the EQ-5D-5L. The estimate was calculated from the study sample, as currently no estimate of the MID if EQ-5D-5L in the general population using the crosswalk tariff is available. The value range for the MID was calculated by multiplying the pooled standard deviation of the EQ-5D-5L utility and VAS scores by 0.2 (to estimate the lower bound) and 0.49 (to estimate the upper bound). This following Cohen’s effect size guidelines [18], where a small effect size between 0.2 SD and 0.49 SD may represent a MID range, and mean differences between arms at or below this range suggests equivalence.

Linear regression was used to assess whether the study arm and a range of background characteristics significantly impacted on EQ-5D-5L utility score (while holding the other indicators included in the model constant). This took the form:

$$ \mathrm{y}=\mathrm{X}\upbeta +\upvarepsilon $$

where y is the utility value, X represents the explanatory sociodemographic variables, and ϵ represents the error term capturing other factors. For each of the utility estimations and the VAS, three models were estimated: study arm and socio-demographic variables (models 1, 4 and 7); study arm and self-reported health variables (models 2, 5 and 8); study arm, demographics and self-reported health variables (models 3, 6 and 9).

EQ-5D-3L data from the respondent’s completion of the first wave YHS questionnaire (between June 2010 and April 2011) were used to compare health differences amongst those who did or did not respond. Differences in scores across the time points were also compared.

## Results

### Response rates

In total, 97 mobile arm and 100 paper arm packs were sent out, with a completion rate of 73 % and 66 % respectively (see Table 1). In the mobile arm, eight respondents returned the phone indicating that they did not consent to take part, and six returned the phone indicating that they had an issue sending the data after completing the EQ-5D-5L. This means that the mobile arm response rate (including completers and those non-consenting or having data sending issues is 87 %). Overall, 12 mobile arm respondents did not complete the EQ-5D-5L or return the phone. One respondent in the mobile arm completed the EQ-5D-5L but did not return the paper questionnaire. In the paper arm, 34 respondents did not complete or return a questionnaire. The demographics and usability results reported here are based on a sample size of 70 for the mobile arm and 66 for the paper arm.

**Table 1:** Response rates across the arms

	Mobile (n,%)	Paper (n,%)
Responder	71 (73.2)	66 (66.0)
Non consent	8 (8.2)	n/a
Data sending issue	6 (6.2)	n/a
Non-responder	12 (12.4)	34 (34.0)

### Demographics

**Fig. 1:**
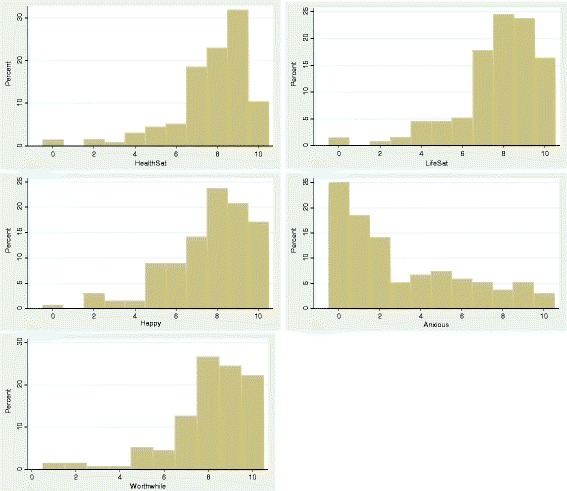
Distribution of wellbeing questions overall

Table 2 displays the demographic characteristics of the two samples. The samples do not significantly differ across any of the characteristics measured. Younger people (aged up to 30) are underrepresented in the sample, with an equal split across the genders. A large majority of the sample are employed and responders are generally well educated (with slightly under half reporting having a university degree or equivalent). Just under half of the sample report at least one long term condition, with a maximum of seven reported. The most common conditions were tiredness (27.9 %), high blood pressure (11.0 %), arthritis (9.5 %), pain (14.0 %) and breathing problems (13.2 %). Overall, respondents reported high levels of health and life satisfaction and feeling worthwhile, a high prevalence of feeling happy, and a low prevalence of feeling anxious (see Fig. 1) with no differences across the arms. The majority of the sample across both arms reported owning a smartphone. In comparison to the first wave YHS data, non-responders report significantly better EQ-5D-3L scores than responders (0.945 vs. 0.896; F_(1,191)_ = 4.25, *p* = 0.04).

**Table 2:** Background characteristics of the two samples

	Mobile (*n* = 70)	Paper (*n* = 66)	Significance
Age			
Mean (SD)	44.5 (12.0)	46.2 (13.3)	0.424
Range	20-64	20-65	
18-30	10 (14.5)	12 (18.2)	0.460
31-50	33 (47.8)	25 (37.9)	
50+	25 (36.2)	29 (43.9)	
Male	34 (48.5)	33 (50.0)	0.868
Marital			
Married/partner	42 (60.0)	42 (63.6)	0.896
Single	17 (24.3)	14 (21.2)	
Other	11 (15.7)	10 (15.2)	
Employment			
Employed	52 (74.3)	53 (80.3)	0.817
Student	4 (5.7)	2 (3.0)	
Retired	6 (8.6)	5 (7.6)	
Not in employment	8 (11.4)	6 (9.1)	
Education past min age	56 (80.0)	46 (70.0)	0.166
Degree	32 (45.7)	32 (48.5)	0.746
Long term condition	32 (45.7)	32 (48.5)	0.883
Number of LTCs			0.417
0	38 (54.3)	34 (51.5)	
1	17 (24.3)	22 (33.3)	
2+	15 (21.4)	10 (15.2)	
Own smartphone	54 (77.1)	48 (72.7)	0.552

### Usability of mobile device

Respondents were positive towards the mobile EQ-5D-5L questionnaire. Overall, 85.5 % agreed that they would complete a questionnaire using a mobile again; 80.0 % agreed that the questionnaire was easy to complete; 88.4 % agreed that the questionnaire could be read on the screen; 85.5 % agreed that they could use the touchscreen; and 87.0 % agreed that the phone was easy to use, simple and clear to understand. Six respondents (all aged over 30) disagreed with all five usability questions, with five of this group owning a smartphone.

### Acceptability of using mobile phones (pencil and paper arm)

In total, 47 respondents in the paper and pencil arm owned a smartphone, with 30 (64 %) indicating that they would use a smartphone to complete questionnaires such as EQ-5D-5L. Of the 18 who did not own a smartphone, six (28 %) reported that they would use phones to complete health questionnaires. Of the 30 respondents who indicated that they would not use a phone to complete a questionnaire, 17 (57 %) were aged over 50 with 11 (37 %) aged between 30 and 50, meaning younger people are more positive towards the use of mobile phones to complete questionnaires than older people.

### Measurement equivalence

#### EQ-5D-5L comparisons across modes

The frequency of respondents endorsing the EQ-5D-5L categories across each of the dimensions does not significantly differ across the modes (Table 3), and there was no missing data across the arms.

**Table 3:** Frequency of responses across the EQ-5D-5L dimensions

Dimension/level	Mobile	Paper	Significance
Mobility			0.10
None	49	57	
Slight	11	5	
Moderate	9	4	
Severe	0	0	
Unable	0	0	
Self-care			0.05
None	62	65	
Slight	6	0	
Moderate	1	1	
Severe	0	0	
Unable	0	0	
Usual activities			0.26
None	54	58	
Slight	9	6	
Moderate	6	2	
Severe	0	0	
Unable	0	0	
Pain/discomfort			0.45
None	26	31	
Slight	34	29	
Moderate	4	5	
Severe	4	1	
Extreme	1	0	
Anxiety/depression			0.88
None	43	44	
Slight	20	17	
Moderate	4	4	
Severe	1	0	
Extreme	1	1	

#### EQ-5D-5L utility comparisons

**Fig. 2:**
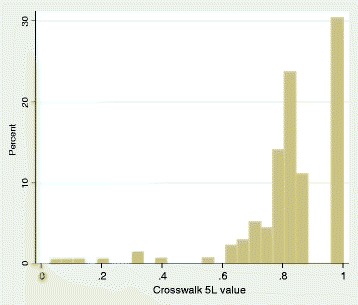
Overall EQ-5D-5L distribution

Figure 2 displays the overall distribution of the EQ-5D-5L utilities estimated using the crosswalk value set [15] . The data is bi modal (which is an artefact of the nature of the EQ-5D-3L UK value set developed by Dolan [14]) and positively skewed. There is also evidence of a ceiling effect (with a substantial proportion of the sample reporting the ‘best’ health state), a common finding in EQ-5D data [19], with less of a ceiling effect in the mobile arm (24 vs. 36 %). The most commonly occurring states were 11111 (30.1 %), 11121 (slight pain or discomfort; 23.5 %), and 11112 (slight anxiety or depression; 9.6 %). That the percentage reporting slight pain or discomfort is generally quite high may demonstrate the increased sensitivity of the EQ-5D-5L descriptive system for this dimension (in comparison to EQ-5D-3L).

The mean EQ-5D-5L utility scores are 0.797 for the mobile arm (range 0.028–1, median 0.837) and 0.845 for the paper arm (range 0.193 to 1, median 0.837). These values are not significantly different (F_(1,133)_ = 2.09, *p* = 0.15). The mean difference between the utility scores is 0.048, and this is just within the lower bound of the estimated MID range (0.047–0.115). Therefore there is the suggestion that the values are equivalent, but the results relating to the MID need to be interpreted with caution due to the instability of the MID estimates carried out on a small sample size.

The mean EQ-5D-5L VAS scores were 80 (Mobile: range 16–100; median 85) and 83 (Paper: range 30–100; median 85) which were not significantly different (F_(1,133)_ = 0.68, *p* = 0.41). The mean difference between the VAS scores is approximately 3, and this is below the lower bound of the estimated MID range (3.5–7.9).

Comparing the paper based original EQ-5D-3L scores from the first YHS wave and the EQ-5D-5L scores from this study demonstrates that correlations between the scores are higher in the paper arm (0.85) than the mobile arm (0.52), but this is difficult to interpret given differences in the descriptive system used and the length of time between administrations.

Table 4 reports regression analysis of the impact of study arm, a range of background variables and self-reported health status on EQ-5D-5L scores. The models suggest that the EQ-5D-5L utility scores are not significantly impacted by study arm, holding demographic characteristics (Models 1 and 4) and self-reported health variables (models 2 and 5) constant. Study arm approaches significance when included in a model with background characteristics to a greater extent than when regressed with self-reported health variables. Model 2 suggests that having a long term condition and low health satisfaction are explanatory factors of EQ-5D score. The study arm does also not impact on VAS score.

**Table 4:** Regression analysis

Parameter	EQ-5D-5L utility score (crosswalk)						EQ-5D-5L utility score (Devlin et al)						EQ-5D-5L VAS score					
	M1		M2		M3		M4		M5		M6:		M7		M8		M9	
	Coef	P	Coef	P	Coef	P	Coef	P	Coef	P	Coef	P	Coef	P	Coef	P	Coef	P
Study arm	0.07	0.05	0.03	0.24	0.03	0.14	0.04	0.05	0.02	0.29	0.02	0.17	3.13	0.27	−1.40	0.44	−0.65	0.69
Gender	0.01	0.75			−0.02	0.39	0.01	0.72			−0.02	0.35	2.09	0.48			−1.30	0.46
Age	−0.00	0.11			−0.00	0.43	−0.00	0.14			0.00	0.43	−0.13	0.27			−0.06	0.40
Marital status	−0.03	0.20			−0.01	0.70	−0.02	0.25			0.00	0.72	−0.86	0.67			1.02	0.38
Employment	−0.02	0.34			0.01	0.26	−0.02	0.20			0.01	0.54	−2.22	0.13			0.17	0.84
Education > min	0.08	0.06			−0.00	0.94	−0.06	0.07			−0.04	0.84	6.19	0.08			−1.10	0.61
Degree	−0.05	0.17			0.01	0.66	−0.04	0.18			0.01	0.50	−4.77	0.12			0.91	0.62
LT condition			−0.05	0.06	−0.04	0.15			−0.03	0.14	−0.02	0.32			1.77	0.38	2.62	0.17
Health sat			0.06	0.00	0.06	0.00			0.05	0.00	0.05	0.00			6.66	0.00	7.14	0.00
Life Sat			−0.00	0.80	0.01	0.50			0.01	0.25	0.00	0.87			−0.33	0.70	0.98	0.23
Happy			−0.01	0.52	−0.01	0.56			−0.00	0.88	0.00	0.97			−0.88	0.29	−0.72	0.35
Anxious			−0.01	0.13	−0.01	0.07			−0.00	0.09	−0.01	0.05			−0.08	0.81	−0.21	0.47
Worthwhile			0.02	0.00	0.01	0.10			0.02	0.00	0.01	0.07			1.99	0.00	0.89	0.14
N	128		129		127		128		129		127		128		129		127	
R^2^	0.10		0.56		0.63		0.10		0.58		0.67		0.08		0.62		0.73	

## Discussion

The results of this study suggest measurement equivalence for EQ-5D-5L when administered across different modes of administration. We found suggested evidence for equivalence of EQ-5D-5L scores across the modes using analysis recommended to test COA equivalence [10], and the findings are in line with past research, not only using EQ-5D [9] but a range of other measures [6, 8, 20]. Coupled with the positive opinions of respondents towards the usability of the mobile phone and the higher response rates, the results lend support to the use of mobile technology to collect data given the advantages of this mode relating to automatic data entry and the increased convenience of flexible completion. We found that older people who were not exposed to using the mobile device were less inclined to say that they would use one, and this is an area where it is important to target in terms of possibly increasing response rates.

In this study a parallel groups design was used to compare the equivalence of the EQ-5D-5L across administration modes. In COA equivalence research, the most widely used methodology in recent is the crossover design, where respondents complete measures using both administration modes [21, 22], and it may be more efficient (and allow for more detailed follow up and usability questions) if this method was used. However using a crossover design was not possible logistically given the methodology used to collect data that involved sending out study materials by post to respondents and asking them the return the completed materials to the study team. This is difficult, as there are many confounding factors relating to the length of time taken to return the first set of materials and then the time taken for the respondent to receive the second set of materials that means that it would be difficult to control experimental issues such as the time between completions. This would limit the inferences that could be drawn from the equivalence analysis carried out, as we could not control for possible changes in health between completions that could differ depending on differences in the time taken to complete both study arms. Therefore a parallel group design was used.

In the guidance paper outlining methods to test the equivalence of COAs across models, a substantially larger sample size per arm than was achieved in this study is recommended (234 per arm). In this study a smaller sample size was possible due to the time available for data collection, and the number of study packs that we would need to be sent to gain a large enough sample (around 700 based on the achieved response rate). We recognise that this is a limitation of this study, but believe that the results still provide an indication of equivalence of the EQ-5D which is a short measure with standardised response options and is therefore amenable to translation as an electronic form and equivalence across administration modes.

There are a range of issues regarding the wider applicability of the findings that need to be considered. Firstly, younger people (who are generally smartphone owners and technology literate) were underrepresented in the sample, and this limits the representativeness of the sample and the wide applicability of the findings. The sample was also generally well educated and in employment which may not represent those recruited to take part in trial, or complete eCOAs in clinical settings. Secondly, respondents who have indicated that they are interested in taking part in a range of research studies were recruited, and this may not reflect other samples (for example in trials) who may not be used to the requirements of research or provide consent. This may mean that the response rate is higher than could be expected. Thirdly, we only required respondents to complete the survey once rather than over time. This means that we could not carry out test-retest reliability analysis, or replicate the trial or health diary aspect of the collection of eCOA that may be an important feature of electronic data collection.

It is also possible that presenting the dimensions page by page in the mobile phone version could influence the results given that the respondent cannot see the full questionnaire in context. This is a general issue for testing equivalence of COA data across modes where page by page completion of electronic versions is recommended (as is the case for EQ-5D-5L). However the results in this study supporting equivalence across modes does not suggest that the format difference was influential. Differences in format could be examined using cognitive debriefing methods and usability testing as suggested by Coons and colleagues [10].

The comparisons that we can make are also limited by the amount of data that we could collect, and also it would be interesting to collect data using a longer generic measures of HRQL such as the SF-12 [21, 22], or other preference based measures such as the SF-6D [23].

The results of this study suggest a range of possible further research studies. Firstly, we have shown equivalence of a paper version with one standard mobile device, but it would be interesting to compare usability across different screen sizes and operating systems (and recent work by Schick-Makaroff and Molzan [24] has shown the feasibility of using iPads to assess quality of life). This is because if COAs and other questionnaires are to be developed as apps (and subsequently released via app stores), for desktops or laptops, or developed for other modes such as smart televisions, then equivalence and usability must be demonstrated across a wide range of devices and screen sizes. This could be done using the qualitative assessment of usability once equivalence has been demonstrated [10], and this has been done for a range of COA [25, 26].

The use of eCOAs is developing quickly and is increasing in a range of settings including clinical trials, Bring Your Own Device (BYOD) settings, and also in routine clinical practice [4]. This study goes some way to supporting the use of EQ-5D-5L in these settings collected using mobile phone technology. Given equivalence is demonstrated, there is also the case for mixing modes to increase response rates in populations who may not have high levels of mobile phone literacy [27].

## Abbreviations

COA: Clinical Outcome Assessment
eCOA: Electronic Clinical Outcome Assessment
ePROM: Electronic Patient Reported Outcome Measure
EQ-5D-3L: euroQol-5 dimension-3 levels
EQ-5D-5L: euroQol-5 dimension-5 levels
FDA: Food and Drug Administration
NICE: National Institute for Health and Care Excellence
ONS: Office of National Statistics
PBAC: Pharmaceutical Benefits Advisory Committee
PDA: Personal Digital Assistant
PROM: Patient Reported Outcome Measure
QALY: Quality Adjusted Life Year
YHS: Yorkshire Health Survey
